# Cost-effective strategies to knock down genes of interest in the retinas of adult zebrafish

**DOI:** 10.3389/fncel.2023.1321337

**Published:** 2024-01-23

**Authors:** Eyad Shihabeddin, Abirami Santhanam, Alexandra L. Aronowitz, John O’Brien

**Affiliations:** ^1^Department of Ophthalmology and Visual Science, McGovern Medical School, The University of Texas Health Science Center at Houston, Houston, TX, United States; ^2^MD Anderson UTHealth Graduate School of Biomedical Sciences, Houston, TX, United States; ^3^University of Houston College of Optometry, Houston, TX, United States

**Keywords:** gene knockdowns, Vivo-Morpholino, siRNA, zebrafish retina, retinal degeneration, regeneration, photoreceptor, retinitis pigmentosa

## Abstract

High throughput sequencing has generated an enormous amount of information about the genes expressed in various cell types and tissues throughout the body, and about how gene expression changes over time and in diseased conditions. This knowledge has made targeted gene knockdowns an important tool in screening and identifying the roles of genes that are differentially expressed among specific cells of interest. While many approaches are available and optimized in mammalian models, there are still several limitations in the zebrafish model. In this article, we describe two approaches to target specific genes in the retina for knockdown: cell-penetrating, translation-blocking Vivo-Morpholino oligonucleotides and commercially available lipid nanoparticle reagents to deliver siRNA. We targeted expression of the PCNA gene in the retina of a P23H rhodopsin transgenic zebrafish model, in which rapidly proliferating progenitor cells replace degenerated rod photoreceptors. Retinas collected 48 h after intravitreal injections in adult zebrafish reveal that both Vivo-Morpholinos and lipid encapsulated siRNAs were able to successfully knock down expression of PCNA. However, only retinas injected with Vivo-Morpholinos showed a significant decrease in the formation of P23H rhodopsin-expressing rods, a downstream effect of PCNA inhibition. Surprisingly, Vivo-Morpholinos were able to exit the injected eye and enter the contralateral non-injected eye to inhibit PCNA expression. In this article we describe the techniques, concentrations, and considerations we found necessary to successfully target and inhibit genes through Vivo-Morpholinos and lipid encapsulated siRNAs.

## 1 Introduction

The growing popularity and diverse usage of high throughput sequencing across different tissues, stages of development, and model organisms has made cell atlases widely available to the public. Genes that are uniquely expressed by a specific cell type are becoming more apparent. Many studies aim to investigate the functional significance of these genes by knocking them down, but techniques that target gene knockdown have to be readily accessible for progress to be made. While many approaches are readily available for mammalian model organisms (Turchinovich et al., 2010; Hatakeyama et al., 2016; Hana et al., 2021) and even larval zebrafish (Schütte et al., 2010; Liang et al., 2022), there is much need for optimization of techniques when it comes to targeted knockdowns in adult zebrafish. Unlike mammalian models, zebrafish have a remarkable capacity throughout their lifespans to regenerate fins, neurons, cardiomyocytes and other tissues when insult is detected (Goldman, 2014; Ail and Perron, 2017; Liu et al., 2018; Santhanam et al., 2020). Identifying the genes responsible for regeneration will allow researchers to translate this knowledge to mammalian models and progress the field of regenerative medicine.

This study offers two different approaches for targeted gene knockdown in adult zebrafish retinas: antisense Vivo-Morpholino oligonucleotides and lipid nanoparticle-complexed siRNA. Morpholinos are a common tool successfully used to knock down expression of genes of interest in zebrafish embryos. However, use of these tools in adult zebrafish is more difficult. Many morpholinos used to target genes in adult retina have been designed with a positively charged lissamine fluorescent molecule as a 3′ end modification (Craig et al., 2010; Thummel et al., 2010, 2011; Gupta et al., 2023), and require electroporation after injection of the morpholino for successful penetrance into cells. Many zebrafish labs do not have an electroporation device readily available, which cost from US $16,000 to $19,000 in 2021, and must rely on alternative strategies. One alternative approach is the use of Vivo-Morpholinos. Vivo-Morpholinos are designed so that a morpholino oligomer is linked to a molecular transporter with eight guanidinium head groups and do not require electroporation for successful penetrance of cells (Morcos et al., 2008). Several studies have successfully used Vivo-Morpholinos for targeted gene knockdown in adult zebrafish (Kim et al., 2010; Hughes et al., 2012; Kyritsis et al., 2012; Kizil et al., 2013; Pfefferli et al., 2014; Chiang et al., 2021); however, none of these studies worked on retina.

While morpholinos typically block translation of mRNA into protein, RNA-Seq identifies differentially expressed transcripts and can reveal transcripts of interest that may not be protein coding (Zhao et al., 2022). Consequently, other strategies are necessary to target some genes. Another popular strategy for targeted knockdown is through the use of siRNAs. SiRNAs utilize the endogenous RISC mechanism to target and degrade mRNA strands of interest (Meister and Tuschl, 2004; Han, 2018). One limitation of using siRNAs for *in vivo* experiments, however, is their successful delivery to the cells of interest (Reischl and Zimmer, 2009; Han, 2018). SiRNA’s have to be packaged and delivered to the cells or tissues of interest without degradation (Reischl and Zimmer, 2009; Wang F. et al., 2016; Han, 2018; Schnell, 2019). One useful strategy for effective delivery to cells is lipid encapsulation of DNA and/or RNA; however, there is variable efficiency in knockdown across different species, age groups, and tissues of interest (Reischl and Zimmer, 2009; Turchinovich et al., 2010; Hatakeyama et al., 2016; Wang F. et al., 2016; Wang F. et al., 2016; Xiao et al., 2018; He et al., 2022). We describe a cost-effective method of siRNA delivery to the zebrafish retina through the utilization of transfection reagents readily available for purchase and use.

In this study, we describe a method for successfully knocking down gene expression in the retina through the use of Vivo-Morpholinos or siRNA. We perform knockdowns in a P23H mutant rhodopsin transgenic zebrafish model developed in our laboratory (Santhanam et al., 2020). This line has been characterized to have continuous degeneration and regeneration of rod photoreceptors throughout adulthood (Santhanam et al., 2020, 2023). Single-cell RNA sequencing and immunohistochemistry have shown PCNA to be a highly expressed gene unique to proliferating cells that eventually become rod photoreceptors in our P23H transgenic zebrafish line (Santhanam et al., 2020, 2023). By targeting PCNA, we are able to directly assess the extent of knockdown as well as whether or not there are any unintended impacts from the technique. We show successful knockdown of PCNA 48 h after injections through immunohistochemistry and qPCR analysis. In eyes injected with Vivo-Morpholinos targeting PCNA, we also see a reduction in the formation of rods, a downstream effect of inhibiting proliferation in progenitor cells. Finally, we identify knockdown of PCNA in the contralateral eyes of the Vivo-Morpholino injected fish, suggesting diffusion of this reagent across the blood-retina barrier.

## 2 Materials and methods

### 2.1 Animal husbandry

Rearing, spawning, and staging of zebrafish (*Danio rerio*) were performed following standard guidelines in the zebrafish community (Westerfield, 2000). Wild-type AB zebrafish were purchased from the Zebrafish International Resource Center (ZIRC; Eugene, OR, USA; RRID:ZIRC_ZL1; ZFIN ID: ZDB-GENO-960809-7). P23H rhodopsin transgenic zebrafish [RRID: uth4tg (AB); ZFIN ID: ZDB-FISH-220323-6] were generated in-house and previously characterized (Santhanam et al., 2020, 2023). All fish were raised on a 14 h light/10 h dark cycle. Both male and female fish between 6 and 12 months of age were used for all experiments in this study. All procedures comply with the U.S. Public Health Service policy on humane care and use of laboratory animals and the NRC Guide for the Care and Use of Laboratory Animals and have been reviewed and approved by the Institutional Animal Care and Use Committees at the University of Texas Health Science Center at Houston under protocol HSC-AWC-21-0040 and at the University of Houston under protocol PROTO202100037.

### 2.2 Oligo preparation

#### 2.2.1 Vivo-Morpholinos

Translation blocking Vivo-Morpholino oligonucleotides were designed and synthesized through GeneTools LLC (Philomath, OR, USA). A control Vivo-Morpholino was designed to target EGFP (Table 1). A PCNA Vivo-Morpholino was designed to target the same sequence as the positively charged lissamine-tagged morpholino used by Thummel et al. (2011; Table 1). Sterile water was used to make a stock concentration of 200 μM for each Vivo-Morpholino. For injections, a working concentration of 40 μM for each Vivo-Morpholino was prepared in sterile filtered phosphate-buffered saline (PBS: 0.01 M phosphate buffer, 0.138 M NaCl, 0.0027 M KCl, pH 7.4) (P3813, Millipore-Sigma). All solutions were stored at room temperature (RT). Stock solutions were stored in the dark when not in use. Working solutions were made fresh for each experiment.

**TABLE 1:** Oligonucleotides used in knockdowns.

Oligo	Target	Sequence	Source	Stock concentration (μM)	Working concentration (μM)
Vivo-Morpholino	PCNA	TTTCTTAGTTTGGAGTAGGAGGAAC	Gene Tools Philomath, OR, USA	200	40
siRNA	PCNA	GTCCAAGACGGTCACACTTAGCATG	IDT Coralville, IA, USA	200	37.5
siRNA	PCNA	AAGACGGTCACACTTAGCATGTCCG	IDT Coralville, IA, USA	200	37.5
siRNA	EGFP	GCATGCATCTCAATTAGTCAGCAAC	IDT Coralville, IA, USA	200	37.5
siRNA	PKCα	CTAAAACCTTGTCCAAAGAAGCAGT	IDT Coralville, IA, USA	200	37.5
siRNA	PKCα	CGCCTTTACATGAAGATTGAGGTGA	IDT Coralville, IA, USA	200	37.5
siRNA	PKCα	CTGCAGTCATTGCACTGACTTCATT	IDT Coralville, IA, USA	200	37.5

#### 2.2.2 siRNA

SiRNAs were designed and synthesized through IDT (Coralville, IA, USA). A control siRNA was designed to target EGFP (Table 1). For PCNA, two siRNAs were generated to target different regions of the PCNA mRNA (Table 1) to increase the likelihood of mRNA degradation (Reischl and Zimmer, 2009; Turchinovich et al., 2010; Han, 2018). Sterile water was used to make a stock concentration of 200 μM for each siRNA. All siRNA’s were aliquoted and stored at −80°C. When designing oligos, the target sequence was run through a BLAST search on either Ensembl or NCBI, and only oligos with no off-targets were selected. For siRNAs, qPCRs were performed to ensure that PCNA transcripts were actually knocked down when compared to the control.

### 2.3 Altogen Nanoparticle *In Vivo* transfection kit

For PCNA siRNA injections, 1.5 μl of each PCNA siRNA (final working concentration of 37.5 μM per siRNA) was combined in a single mixture with 5 μl of Altogen Nanoparticle *In Vivo* transfection reagent (Catalog # 5030, Las Vegas, NV, USA). For control siRNA injections, 3.0 μl of EGFP siRNA (final working concentration of 75 μM) was combined in a single mixture with 5 μl of Altogen Nanoparticle *In Vivo* transfection reagent. Each mixture was incubated at RT for 20 min before adding 1 μl of Altogen Nanoparticle *In Vivo* transfection enhancer. This solution was incubated for 5 min at RT. Working solutions were made fresh for each experiment.

### 2.4 Mirus *Trans*IT TKO kit

For PCNA siRNA injections, 1.6 μl of each PCNA siRNA (final working concentration of 40 μM per siRNA) was combined in a single mixture with 4.8 μl of Mirus TKO *Trans*IT-TKO Reagent (Catalog # 2154, Madison, WI, USA). For control siRNA injections, 3.2 μl of EGFP siRNA (final working concentration of 80 μM) was combined in a single mixture with 5 μl of Mirus TKO *Trans*IT-TKO Reagent. Each mixture was incubated at RT for 20 min before injections. Working solutions were made fresh for each experiment.

### 2.5 Intravitreal injections

**FIGURE 1:**
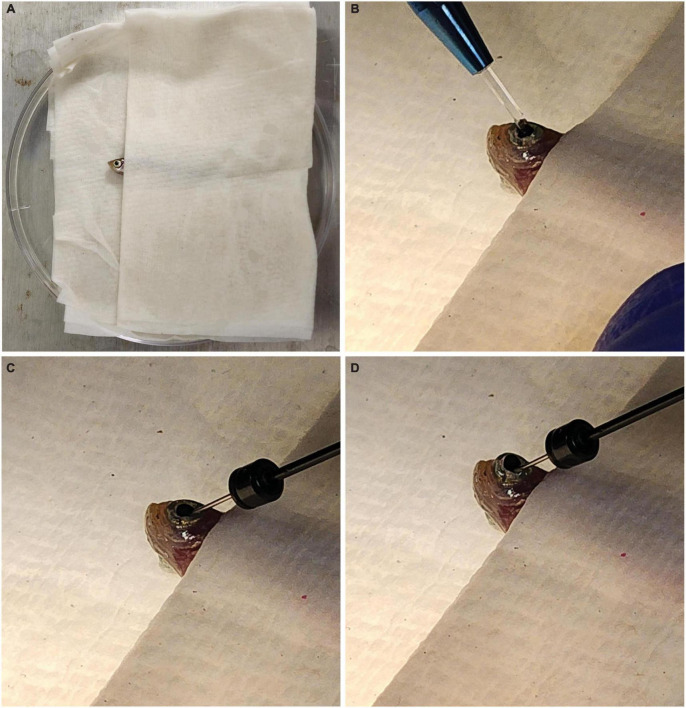
Fish setup for intravitreal injections. **(A)** Fish are placed in a moist towel so that only their heads are exposed for intravitreal injections after being anesthetized. **(B)** A sapphire blade is used to make an incision between the pupil and outer edge of the iris. **(C)** A blunt syringe is used to remove 1.0 μl of vitreous humor from the eye. **(D)** A total of 1.0 μl of the solution of interest is injected at the incision site.

Each fish was anesthetized in 0.02% Tricaine/MS222 until it was no longer responsive to being picked up with a plastic spoon. A towel was then dipped in fish water and placed inside a Petri dish lid. The fish was placed inside the towel so that only its head was exposed (Figure 1A). Under a microscope, a sapphire blade (World Precision Instruments, Sarasota FL, USA; Catalog #504072) was used to make an incision between the pupil and the outer edge of the iris (Figure 1B). A 32 gauge blunt NEUROS Syringe (Stoelting, Wood Dale, IL, USA; Catalog #53493) was inserted at the site of incision and 1.0 μl of vitreous humor was removed from the eye and expelled from the needle (Figure 1C). We found this removal necessary for the solutions to make contact with the retina and enter the cells. When no vitreous humor was removed, knockdown was not seen. A total of 1.0 μl of either PCNA Vivo-Morpholino, Control Vivo-Morpholino, PCNA siRNA, or Control siRNA was then injected at the incision site of the eye (Figure 1D). The fish was replaced in an isolated tank and kept under observation until it was collected 48 h post injection (hpi). A video of this procedure can be found in Supplementary Movie 1.

### 2.6 Tissue collection and preparation

At 48 hpi, each fish was collected and euthanized in 0.15% Tricaine/MS222. Eyeballs were excised from the head and fixed in 9:1 95% EtOH:32% formaldehyde (PFA) (Catalog #15714-S, Electron Microscopy Sciences, Hatfield, PA, USA) for 30 min. Eyeballs were then given 3 × 5 min washes in 1× PBS before being placed in 25% sucrose overnight. The next day, eyeballs were embedded in Tissue-Tek O.C.T. compound (Sakura Finetek, Torrance, CA, USA) and kept frozen at −80°C until they were cryosectioned. Tissue slices were 12 μm thick.

### 2.7 Immunohistochemistry

Slides from the dorsal, ventral, and middle of the retina were used for immunohistochemistry by incubation in (i) blocking solution with 0.3% Triton X-100, 5% of the serum of the species in which the secondary antibody was generated (either Donkey Serum or Goat Serum; Jackson ImmunoResearch; West Grove, PA, USA) in PBS for 1 h at RT; (ii) primary antibody diluted in PBS, 0.1% Triton-X100, and 5% serum overnight at RT; (iii) fluorescent secondary antibody, diluted as the primary antibody, for 1 h at RT. For nuclear counterstaining, retinal sections were mounted in Vectashield with DAPI (H-1000; Vector Laboratories; Burlingame, CA, USA) and coverslipped. The primary and secondary antibodies used in this study are listed in Table 2. Images were taken using Zeiss LSM 800 confocal microscope with a 40× objective lens (Thornwood, NY, USA).

**TABLE 2:** Information about antibodies used in study.

Antibody	Host	Antigen	Source	Catalog number	RRID	Dilution
Flag-DDK	Ms	DYKDDDDK	OriGene**** Rockville, MD, USA	TA50011	AB_2622345	1:200
PCNA	Rb	Synthetic peptide corresponding to Human PCNA aa 200 to the C-terminus	Abcam**** Cambridge, MA, USA	Ab18197	AB_444313	1:100
PKCα	Rb	Synthetic peptide corresponding to Human PKCα aa 373-672 to the C-terminus	Santa Cruz Biotechnology**** Dallas, TX, USA	SC-10800	AB_2168560	1:100
Cy3	Gt	Goat Anti-Mouse IgG Fc 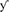 subclass 2a specific	Jackson ImmunoResearch**** West Grove, PA, USA	115-165-206	AB_2338695	1:500
Alexa Fluor 488	Dk	Donkey Anti-Rabbit IgG	Jackson ImmunoResearch**** West Grove, PA, USA	711-545-152	AB_2313584	1:500

### 2.8 Quantification

A maximum intensity projection of each image taken with a 40× objective lens at 1× confocal zoom was imported into FIJI (ImageJ 1.53t) where the image was cropped to 1024 × 376 pixel region of interest that included the outer nuclear layer (ONL) and photoreceptor outer segment (OS) layers. The image was then split by each channel (i.e., red, green, and blue). For red (Flag-tag) and green (PCNA or PKCα) channels, a threshold was adjusted to capture only the signal. After applying the threshold, a binary image mask showed the signal as white and background as black. A histogram that quantified the number of black pixels and white pixels found in the region of interest was then generated and saved in Excel 2016. For each image, the ratio of white pixels (signal) to total pixels was calculated. In GraphPad Prism 9, a two-tailed *t*-test was performed to compare signals between the control and injected fish for knockdown approach. A *p*-value <0.05 was considered significantly different. To further validate PKCα knockdowns, the length (in pixels) of PKCα positive axons was recorded for each image. In GraphPad Prism 9, a two-tailed *t*-test was performed to compare PKCα axon lengths between the control and injected fish. A *p*-value <0.05 was considered significantly different.

### 2.9 qPCR

At 48 hpi, 4 fish for each condition (EGFP control or PCNA) were collected and euthanized in 0.15% Tricaine/MS222. Eyeballs were excised from the head and retinas extracted from the eyecup and placed in a 1.5 ml Eppendorf tube. Each retina was then flash frozen with liquid nitrogen and crushed with a disposable pestle (BPI-4030-PB; Capitol Scientific). After about 30 s, 700 μl of PureZol (7326880; Bio-Rad) was added to the retina sample. Retinas were further homogenized for another 30 s until no more clumps were seen. Samples were then incubated at RT for 5 min. After incubation, samples were processed through the Aurum Total RNA Fatty and Fibrous Kit (732-6830; Bio-Rad) to collect purified RNA. The collected RNA samples were then processed through iScript Reverse Transcription Supermix for RT-qPCR (1708841; Bio-Rad) to obtain 300 ng of cDNA per sample. Throughout this process, one sample from a PCNA injected fish was lost. Following the iTaq Universal SYBR Green Supermix (1725120; Bio-Rad) protocol, master mixes for each sample and primer pairs (Table 3) were prepared for qPCR. The qPCR reaction was run in CFX Opus 96 Real-Time PCR System from Bio-Rad. The thermocycling parameters were: step 1: 95°C for 2 min, step 2: 95°C for 30 s, step 3: 62°C for 1 min, step 4: 68°C for 30 s with steps 2–4 repeating 39 times. Finally the thermocycler performed a melt curve from 65 to 95°C at 0.5°C increments, with a recording at 5 s/increment. Results were then exported through Bio-Rad’s CFX Maestro as a CSV file (Supplementary Table 1). For each sample in the qPCR, a triplicate was measured and averaged together. GAPDH was used as a housekeeping gene and a PCNA qPCR target was used for measurements (primer sequences in Table 3).

**TABLE 3:** Oligonucleotides used for qPCR.

Target	Orientation	Sequence
PCNA	Forward	CGACAAGGAGGATGAAGCGGTAACA
PCNA	Reverse	GTACTCAACCACTAGTGGAATATC TGCGGAC
GAPDH	Forward	ATGACCCCTCCAGCATGA
GAPDH	Reverse	GGCGGTGTAGGCATGAAC

## 3 Results

### 3.1 PCNA expression

**FIGURE 2:**
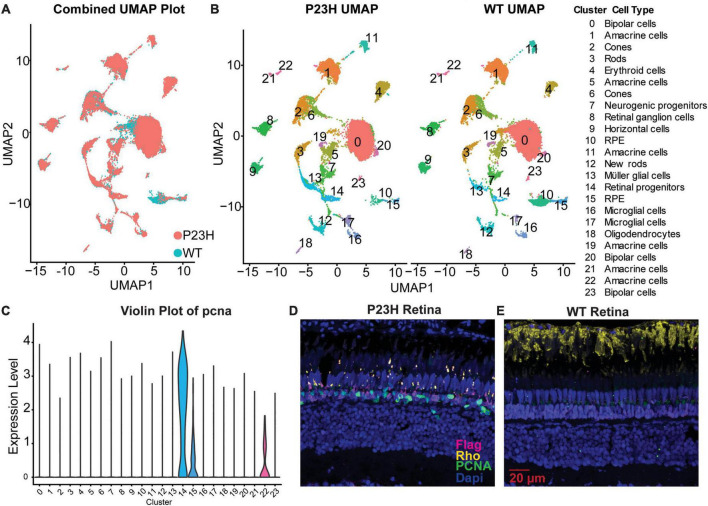
PCNA expression across cell types in zebrafish retina. **(A)** P23H and WT single-cell RNA-sequencing data integrated into a single UMAP projection overlaying WT (teal) and P23H (orange) cells. **(B)** Separate UMAP projections of the 24 unique clusters identified in the cluster analysis with cell identities. **(C)** Violin plot of *pcna* expression shows predominant expression in cluster 14-retinal progenitor cells. **(D)** Immunostaining for Flag tag (magenta), PCNA (green), and rhodopsin (yellow) in P23H adult retina. **(E)** Immunostaining for Flag tag (magenta), PCNA (green), and rhodopsin (yellow) in WT adult retina.

We previously performed single-cell RNA sequencing (SC RNA-Seq) analysis on retinas from adult WT and P23H transgenic zebrafish (expressing Flag-tagged P23H mutant rhodopsin in rods) to examine how the retinal environment changed during retinal degeneration and regeneration (Santhanam et al., 2023). WT and P23H SC RNA-Seq data were integrated to allow for cells in the same cluster to be compared between conditions (Figure 2A). We identified 24 different clusters and many transcriptional differences between WT and P23H within each cluster [Figure 2B; data from Santhanam et al. (2023)]. Analysis of the integrated data identified that PCNA expression was predominantly found to be in cluster 14, retinal progenitor cells, and at a much lower level in a few other cell types (Figure 2C). Immunostaining of retinal slices collected from WT and P23H retina showed PCNA (green) expressed in P23H retina and rarely in the WT retina (Figures 2D, E). Furthermore, expression of PCNA was primarily found in the ONL where new rods are continuously being generated by progenitor cells (Santhanam et al., 2020). Immunostaining for the Flag tag (DDK – magenta, Figures 2D, E) was only present in the P23H line and revealed small, deformed rod photoreceptors in the ONL, in keeping with the retinal degeneration phenotype of this transgenic line. Unlike native rhodopsin (yellow), which is present almost exclusively in rod OSs in wild type retina, Flag-tagged P23H rhodopsin is delocalized over the entire surface of the rods, labeling the somas and synaptic terminals as well as the very small OSs (Figures 2D, E).

### 3.2 Injection technique

To explore targeted gene knockdown in adult zebrafish retina, Vivo-Morpholino oligos or siRNA were injected intravitreally into adult eyes. The setup for all injections was the same. Zebrafish were anesthetized in Tricaine (MS222) and then placed between layers of a folded wet towel, exposing only the side of the head with the eye designated for injection (Figure 1A). An incision with a sapphire blade was made by hand at a 45° angle between the pupil and the outer edge of the iris (Figure 1B). After the incision, a blunt end Hamilton syringe mounted on a micromanipulator angled at 45° was used to enter the eye at the point of incision and remove 1 μl of vitreous humor (Figure 1C). The same syringe was then used to inject the solution mixture (either Vivo-Morpholino or encapsulated siRNA) into the eye (Figure 1D). A video of this procedure can be viewed Supplementary Movie 1. The fish was then replaced in water for recovery. After 48 hpi, fish were collected for analysis.

### 3.3 Injected eyes

#### 3.3.1 Vivo-Morpholinos

**FIGURE 3:**
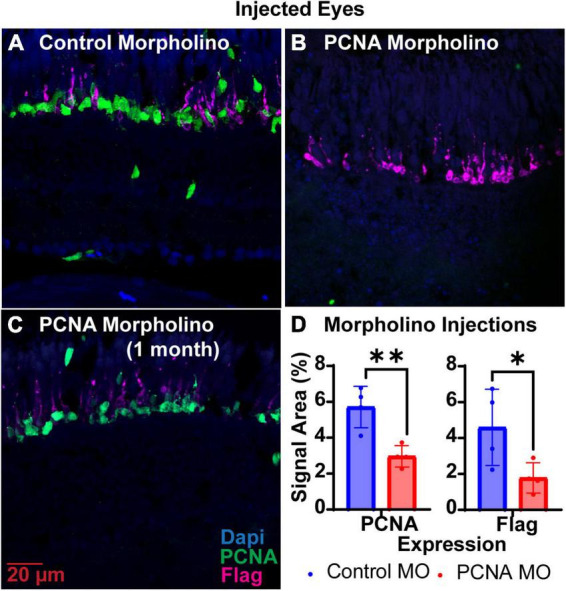
PCNA Vivo-Morpholino knockdown in retinas of injected eyes. **(A)** PCNA and Flag tag expression in retinas of eyes injected with control Vivo-Morpholino. **(B)** PCNA and Flag tag expression in retinas of eyes injected with fresh stock solution of PCNA Vivo-Morpholino. **(C)** PCNA and Flag tag expression in retinas of eyes injected with 1 month old stock solution of PCNA Vivo-Morpholino. **(D)** Quantitative analysis comparing PCNA and Flag tag expression between eyes injected with control Vivo-Morpholino or PCNA Vivo-Morpholino reveal a significant decrease in both PCNA and Flag tag expression (PCNA *p* = 0.0056; Flag *p* = 0.0489). *p < 0.05, **p < 0.01.

Translation blocking Vivo-Morpholinos were generated to target the translation start sites of PCNA (target of interest) and EGFP (control) (Table 1). Each zebrafish was injected in one eye with either the PCNA Vivo-Morpholino or control Vivo-Morpholino. Both eyeballs, labeled as injected eye and contralateral eye, were collected 48 hpi. Slides collected were stained for PCNA (green), Flag-DDK (magenta), and Dapi (blue). For each condition, a *n* of four fish was used. As PCNA and Flag-DDK are continuously and predominantly expressed in the ONL and photoreceptor OS of the P23H non-injected zebrafish eye (Figure 2D), quantifications were determined by the area percentage of signal expression per total area of the ONL and OS. The control Vivo-Morpholino injected eyes had a mean PCNA signal expression of 5.71% ± 1.16% (Figures 3A, D). The PCNA Vivo-Morpholino injected eyes showed a significant reduction of PCNA signal (Figure 3B) with a mean signal expression of 2.97% ± 0.6% (*p* = 0.0056, unpaired *t*-test; Figure 3D). The same tissue slices were also co-stained with Flag-DDK antibody. The Flag tag is unique to the P23H mutated rhodopsin (Figure 2D) and is not seen in WT fish (Figure 2E; Santhanam et al., 2020). The control Vivo-Morpholino injected eyes had a mean Flag tag signal expression of 4.59% ± 2.12% (Figures 3A, D). The PCNA Vivo-Morpholino injected eyes showed more than 50% reduction of Flag tag signal (Figure 3B) with a mean signal expression of 1.78% ± 0.84% (Figure 3D). This change was statistically significant (*p* = 0.0489, unpaired *t*-test), indicating that PCNA knockdown reduced expression of the mutant rhodopsin transgene.

#### 3.3.2 SiRNAs

**FIGURE 4:**
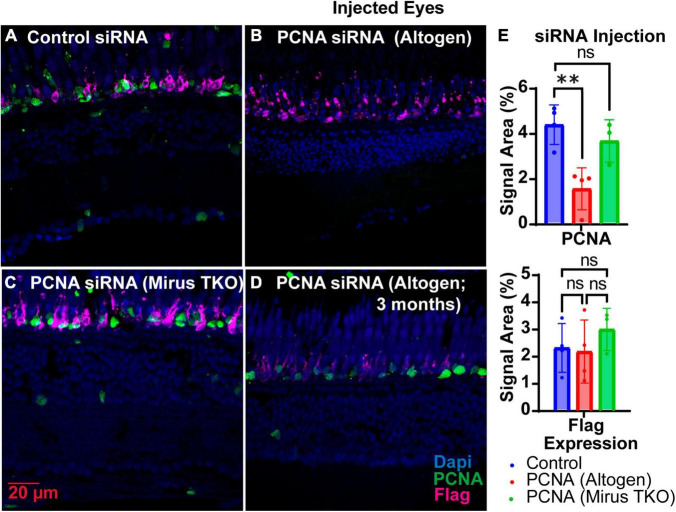
PCNA siRNA knockdown in retinas of injected eyes. **(A)** PCNA and Flag tag expression in retinas of eyes injected with control siRNA encapsulated through Altogen lipid nanoparticles. **(B)** PCNA and Flag tag expression in retinas of eyes injected with PCNA siRNA encapsulated through Altogen lipid nanoparticles. **(C)** PCNA and Flag tag expression in retinas of eyes injected with siRNA encapsulated through Mirus TKO Kit. **(D)** PCNA and Flag tag expression in retinas of eyes injected with PCNA siRNA encapsulated through 3 month old Altogen lipid nanoparticles. **(E)** Quantitative analysis comparing PCNA and flag tag expression between eyes injected with control siRNA and PCNA siRNA reveal a significant decrease in PCNA expression (*p* = 0.043) but not Flag tag expression (*p* = 0.8612). PCNA is green, Flag tag expression is magenta, and Dapi is blue. **p < 0.01, ns = not significant.

Two siRNAs were generated through IDT’s siRNA design tool to target different regions of PCNA mRNA, while one siRNA was designed to target EGFP as a control (Table 1). Each zebrafish was injected in one eye with either a solution containing both PCNA targeting siRNAs or the control siRNA. Both eyeballs, labeled as injected eye and contralateral eye, were collected 48 hpi. Slides collected were stained for PCNA (green), Flag-DDK (magenta), and Dapi (blue). For each condition, a *n* of four fish was used. The Altogen nanoparticle kit yielded a significant knockdown of PCNA expression. The control siRNA injected eyes had a mean PCNA signal expression of 4.25% ± 0.86% (Figures 4A, E). The PCNA siRNA injected eyes showed on average about 65% reduction in PCNA signal in the ONL, with a mean signal expression of 1.47 ± 0.91 (*p* = 0.00430, unpaired *t*-test; Figures 4B, E). The same tissue slices were also co-stained with Flag-DDK antibody. The control siRNA injected eyes had a mean Flag tag signal expression of 2.73% ± 1.1% in the ONL (Figures 4A, E). The PCNA siRNA injected eyes showed no significant reduction, with an average expression of 2.56% ± 1.4% (*p* = 0.8612, unpaired *t*-test; Figures 4B, E). As additional validation of efficacy of siRNA knock down, we examined PCNA transcript levels by qPCR and found that relative expression of PCNA was significantly lower in the PCNA siRNA conditions compared to the control EGFP conditions (*p*-value = 0.0347, unpaired *t*-test). A Mirus TKO Transfection Reagent kit was also used to make the same injections; however, it yielded no significant knockdown of PCNA or Flag-tagged mutant rhodopsin (Figures 4C, E).

**FIGURE 5:**
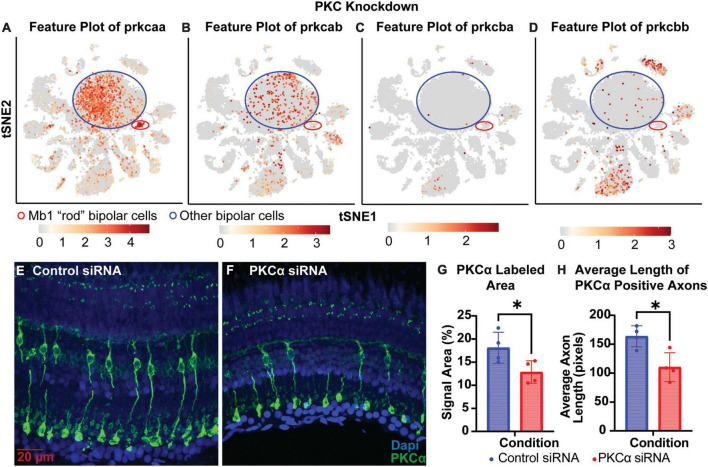
PKCα knockdown in retinas of injected eyes. **(A–D)** Feature plots of PKC isoform expression in integrated P23H and WT single-cell RNA-sequencing data in tSNE projections. The Mb1 “rod” bipolar cell cluster expressing high amounts of *prkcaa* is circled in red, while a large mixed cluster of other bipolar cell types is circled in blue. **(E)** PKCα immunolabeling in retinas of eyes injected with control siRNA encapsulated through Altogen lipid nanoparticles revealing well-labeled Mb1 “rod” bipolar cells. **(F)** PKCα immunolabeling in retinas of eyes injected with PKCα siRNA encapsulated through Altogen lipid nanoparticles. **(G)** Quantification analysis comparing PKCα expression between eyes injected with control siRNA and PKCα siRNA revealed a significant decrease in PKCα expression (*p* = 0.0439). **(H)** Quantification of the average length of PKCα positive axons reveal a significant decrease in lengths (*p* = 0.0139). PKCα is green and Dapi is blue. *p < 0.05.

To test whether knockdowns could be achieved in cells that have terminally differentiated and exited the cell cycle, we generated siRNA to target PKCα. PKCα is abundant in bipolar cells, providing a readily visible marker for the “rod” bipolar cell, and is important for normal cell signaling in the retina (Haug et al., 2019). Analysis of single-cell RNA sequencing data from Zf retina (data from Santhanam et al., 2023)^1^ revealed that *prkcaa* is expressed predominantly in bipolar cells, but is also the only isoform highly expressed in rod bipolar cells (Figure 5A). *Prkcab* is expressed predominantly in bipolar cell populations (Figure 5B). PKCß isoforms *prkcba* and *prkcbb* are expressed predominantly in cell types other than bipolar cells (Figures 5C, D). For this study, we decided to target *prkcaa* and evaluate efficacy with an anti-PKCα antibody (Table 2). Knockdown of *prkcaa* resulted in a 33% reduction in PKC expression (Figures 5E–G; *p* = 0.0439). While still significant, this low degree of knockdown may be in part due to the antibody’s lack of specificity to discriminate among the PKC isoforms (Haug et al., 2019), whereas the siRNA used was specific to *prkcaa* (Table 1). To further validate knockdown, we measured the average length of PKC positive axons and found the average length of PKC positive axons was reduced by about 33% (Figures 5F, H; *p* = 0.0139). Overall, our data show that targeted gene knockdowns can be achieved in both actively dividing and terminally differentiated cell types in the retina.

#### 3.3.3 Efficacy of oligo preparations

The shelf life of both Vivo-Morpholinos and Altogen nanoparticles kit was much shorter than anticipated. Stocks of the Vivo-Morpholinos were made up the day before the first set of experiments. For each set of experiments, working solutions were made fresh the day of injections. After 1 month, the stock solution had precipitates that would not go back into solution after heating and the Vivo-Morpholino was no longer effective (Figure 3C). We have found that injections within the first 2 weeks of stock solution preparation have yielded the best results. The Altogen nanoparticles kit has a reported shelf life of 6 months; however, we saw no knockdowns using the kit after 3 months (Figure 4D).

### 3.4 Non-injected contralateral eyes

#### 3.4.1 Vivo-Morpholinos

**FIGURE 6:**
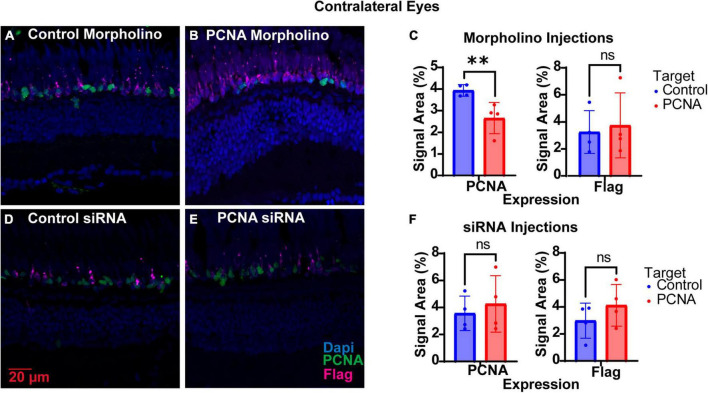
PCNA knockdown in retinas of non-injected contralateral eyes. **(A)** PCNA and Flag tag expression in retinas contralateral to eyes injected with control Vivo-Morpholino. **(B)** PCNA and Flag tag expression in retinas contralateral to eyes injected with PCNA Vivo-Morpholino. **(C)** Quantitative analysis comparing PCNA and Flag tag expression between retinas contralateral to eyes injected with control Vivo-Morpholino and PCNA Vivo-Morpholino reveal a significant decrease in PCNA expression (*p* = 0.0161) but not Flag tag expression (*p* = 0.7433). **(D)** PCNA and Flag tag expression in retinas contralateral to eyes injected with control siRNA encapsulated in Altogen lipid nanoparticles. **(E)** PCNA and Flag tag expression in retinas contralateral to eyes injected with PCNA siRNA encapsulated in Altogen lipid nanoparticles. **(F)** Quantitative analysis comparing PCNA and Flag tag expression between retinas contralateral to eyes injected with control siRNA and PCNA siRNA reveal no significant difference between conditions (PCNA *p* = 0.5887; Flag tag *p* = 0.3037). PCNA is green, Flag tag expression is magenta, and Dapi is blue. **p < 0.01, ns = not significant.

The non-injected contralateral eyes were also collected from each Vivo-Morpholino injected fish, initially to serve as an internal control. Retinas collected were fixed, stained with PCNA (green), Flag-DDK (magenta), and Dapi (blue) and quantified as for the injected eyes. To our surprise, we discovered that non-injected contralateral eyes of fish injected with the PCNA Vivo-Morpholino showed significant knock down of PCNA expression. The non-injected contralateral eyes of the fish injected with the control Vivo-Morpholino showed an average PCNA expression of 3.93% ± 0.27% (Figures 6A, C). The non-injected contralateral eyes of the fish injected with the PCNA Vivo-Morpholino showed a 40% reduction in PCNA expression, with an average expression of 2.66% ± 0.72% (*p* = 0.0161, unpaired *t*-test; Figures 6B, C). While PCNA was knocked down, labeling with the Flag-DDK antibody was not changed. The non-injected contralateral eyes of the fish injected with the control Vivo-Morpholino showed an average Flag tag signal of 3.25% ± 1.58% while the non-injected contralateral eyes of the fish injected with the PCNA Vivo-Morpholino showed an average Flag tag signal of 3.74% ± 2.4% (*p* = 0.7433, unpaired *t*-test; Figures 6A–C).

#### 3.4.2 SiRNA injections

**FIGURE 7:**
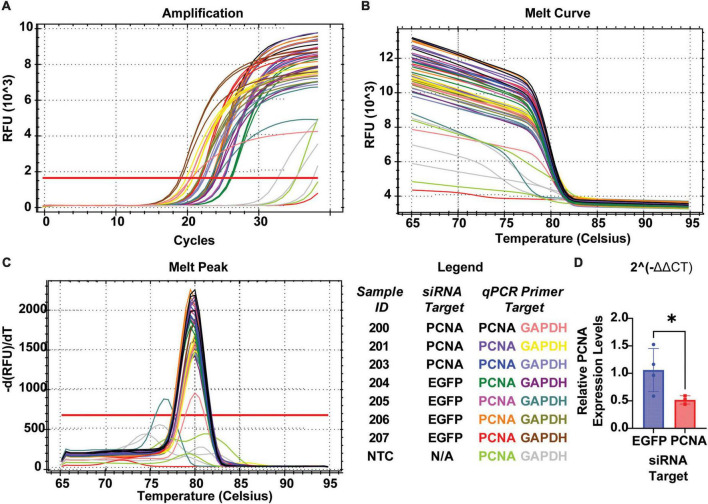
QPCR analysis of PCNA knockdown in retinas of eyes injected with siRNA encapsulated in Altogen lipid nanoparticles. **(A)** Amplification curves for each sample run in qPCR. **(B)** Melt curve for each sample run in qPCR. **(C)** Melt peaks of each sample run in qPCR. **(D)** Relative expression of PCNA through 2^– Δ^ ^Δ^ ^CT^ analysis reveals a significant decrease in PCNA expression when compared to control (*p* = 0.0347). *p < 0.05.

The non-injected contralateral eyes were also collected from each siRNA injected fish. Slides collected were fixed, stained, and quantified similarly as the injected eyes with PCNA (green), Flag-DDK (magenta), and Dapi (blue). The non-injected contralateral eyes of the fish injected with the control siRNA showed an average PCNA expression of 3.57% ± 1.28% (Figures 6D, F). The non-injected contralateral eyes of fish injected with PCNA siRNA showed an average PCNA expression of 4.27% ± 2.09% (Figures 6E, F). This was not significantly different than the control (*p* = 0.5887, unpaired *t*-test). Likewise, Flag tag expression was unchanged in contralateral eyes of siRNA injected fish. The non-injected contralateral eyes of fish injected with control siRNA showed an average Flag tag expression of 2.99% ± 1.3% while fish injected with PCNA siRNA showed an average Flag tag expression of 4.12% ± 1.54% (*p* = 0.3037, unpaired *t*-test; Figures 6D–F). Retinas from siRNA injected fish were also collected and analyzed through qPCR. For quality control, the amplifications, melt curves, and melt peaks of each sample were recorded and distinct from the no template controls (NTC; Figures 7A–C). The CT value of each sample was below 28, while the CT value of each NTC was either not detected or above 34, indicating no contamination in the samples (Supplementary Table 1). ΔCT values were then normalized to the control and plotted as a function of 2^–ΔΔ^*^CT^* (Figure 7D). Relative expression of PCNA was significantly lower in the PCNA siRNA conditions compared to the control EGFP conditions (*p*-value = 0.0347).

## 4 Discussion

High throughput sequencing has made targeted gene knockdown a more common approach in identifying the roles of several differentially expressed genes in cells of interest. While optimized protocols and reagents are widely available for *in vitro* knockdowns or *in vivo* knockdowns in mammalian models, approaches are still limited in the zebrafish model (Thummel et al., 2006, 2011; Kim et al., 2010). We have identified and discuss two different approaches for targeted gene knockdown in zebrafish retina: the use of Vivo-Morpholinos and siRNA delivery through lipid-nanoparticle encapsulation. For these experiments, we have chosen to knock down proliferating cell nuclear antigen (PCNA). PCNA is a well characterized gene found to express late in the G1 phase and throughout S phase of the cell cycle (Kurki et al., 1987; Strzalka and Ziemienowicz, 2011). In the retina, where neurons terminally differentiate and exit the cell cycle after development, PCNA expression is unique to newly forming cells and has provided great insight to cells involved in zebrafish retina regeneration (Fimbel et al., 2007; Kassen et al., 2007; Goldman, 2014; Santhanam et al., 2020). Our P23H rhodopsin transgenic zebrafish show continuous degeneration and regeneration of rod photoreceptors with constant expression of PCNA predominantly in the ONL of the retina (Figures 2C, D), making PCNA an ideal target for testing knockdowns (Santhanam et al., 2020).

Vivo-Morpholinos were designed to target the translation start sites of PCNA mRNA. Zebrafish were injected in one eye with the Vivo-Morpholino, and both injected and contralateral non-injected eyes were collected for immunohistochemistry 48 hpi. On average, there was a 50% reduction of PCNA expression throughout the ONL of the retina in the injected zebrafish eye (Figures 3B, D). Knockdown was seen throughout the entire retina and was not confined to a specific region or depth. Furthermore, flag-tag expression was knocked down by more than half when compared to the control (Figures 3B, D). The flag-tag is expressed at the end of the rhodopsin protein carrying the P23H mutation and is continuously expressed in rod photoreceptors (Santhanam et al., 2020). We presume that the reduction of Flag-tag expression is a result of prevention of new rod formation when proliferation of progenitor cells was halted, along with ongoing degeneration of existing rods. Vivo-Morpholinos demonstrate effective knockdown of the target gene long enough for pathways downstream of the affected gene to be impacted.

SiRNAs were designed to target and degrade mRNA transcripts of the gene of interest. On average, there was a 75% reduction in PCNA expression throughout the entirety of the retina when siRNAs were encapsulated in the Altogen nanoparticles kit (Figures 4B, E), and a 33% reduction in PKCα expression when targeting non-dividing bipolar cells (Figures 5F, G). Successfully packaging multiple siRNAs allows for efficient targeting of transcripts of a gene for degradation. Furthermore, different genes can be targeted simultaneously to study a specific pathway or compensatory mechanism. While siRNAs demonstrated effective knockdown of the target gene, we did not observe any impact on pathways downstream of the affected gene (i.e., reduction of new rod formation as measured by Flag-tag expression). We believe this may be due to the kinetics by which inhibition occurs. Vivo-Morpholinos bind to RNA directly and inhibit translation. SiRNAs must utilize the endogenous RISC mechanism to target and degrade mRNAs of interest (Meister and Tuschl, 2004; Han, 2018)). As such, we believe that it may take longer than the 48 h timepoint chosen for this study to be able to successfully see downstream affects through siRNA inhibition. The kinetics for siRNA inhibition varies depending on the siRNA design and how many different siRNAs are used in conjunction (Krzysztoń et al., 2019). The time point at which downstream effects become apparent will vary for each siRNA designed and should be evaluated on an individual basis, if desired. The siRNA knockdown approach is very cost-effective when trying to screen multiple candidate genes found in RNA-Seq data. Furthermore, this technique has the potential to target long-non-coding RNA and other transcripts that may not be protein coding.

Surprisingly, we saw knockdown of PCNA expression in the non-injected contralateral eyes under the Vivo-Morpholino condition (Figure 6B). Such a phenomenon indicates that the Vivo-Morpholino is able to successfully exit the injected eye and eventually enter the contralateral eye. Nanoparticle trafficking in the eye for mammalian models suggest that in order for this phenomenon to happen, the Vivo-Morpholino permeated through the blood–retina barrier (BRB) and entered the blood supply (Swetledge et al., 2021). Upon entry, the Vivo-Morpholino circulated systemically until it returned to the retina of the contralateral eye (Swetledge et al., 2021). While GeneTools has not tested the BRB directly, they have shown that Vivo-Morpholinos can pass through the blood–brain barrier (BBB) and reach the brain when systemically administered in mammalian models, although, effectiveness of the Vivo-Morpholino is usually lost (Morcos et al., 2008). As such, it is possible that the Vivo-Morpholino made its way into the contralateral retina through an alternative, more direct, path. Future studies will have to investigate the phenomenon further.

It should be noted that both Vivo-Morpholinos and lipid encapsulation delivery of siRNAs have given us issues with long term storage. We stored the Vivo-Morpholinos at RT in the dark, following Gene-Tools recommendations; however, Vivo-Morpholinos would progressively lose efficacy as the stock solution aged and precipitated out of stock solution within a month. Over the course of the injections, the Altogen transfection reagent would form precipitates. After about 3 months, the Altogen transfection reagent had to be replaced. When using either approach, one should be mindful of these factors in case knockdown efficiency changes when replicating an experiment.

Another area to note is how our injections were performed. The incision with the sapphire blade was made by hand each time. Physical damage to the eye, and especially to the retina, can cause a proliferative and inflammatory response separate from the phenomenon one may be trying to study. One needs to make sure the proper controls are in place to account for any confounding factors that may arise. In our model, PCNA is normally expressed almost exclusively in the ONL; however, when acute damage to the retina is detected, Müller glial cells become activated and progenitor cells expressing PCNA form in the inner nuclear layer (INL) (Thummel et al., 2011; Goldman, 2014; Hamon et al., 2016). To mitigate the effects of acute damage and focus on whether knockdown was successful, we quantified PCNA expression specifically in the ONL and OS. Furthermore, 1.0 μl of vitreous humor was removed prior to injection of either the Vivo-Morpholino or the lipid-nanoparticle complexed siRNA. This allowed for a more direct contact between the injection solution and the retina. We found that not removing vitreous humor greatly reduced knockdown efficacy.

Many labs are utilizing high throughput sequencing techniques like single-cell RNA sequencing to identify differentially expressed genes unique to a cell type or cluster. Techniques available for adult zebrafish to test how knockdown of these genes affect specific pathways or mechanisms are limited. We have shown two methods that efficiently knock down a gene of interest long enough for changes in downstream pathways to be visualized. Although there can be some variability and knockdowns should be optimized for each gene of interest, both techniques provide labs with no prior experience in knockdowns an efficient approach to screen and test genes identified as differentially expressed via sequencing.

## Data availability statement

The data presented in the study are deposited in GEO, https://www.ncbi.nlm.nih.gov/geo/ accession number GSE234435.

## Ethics statement

The animal study was approved by the Institutional Animal Care and Use Committee, University of Houston. The study was conducted in accordance with the local legislation and institutional requirements.

## Author contributions

ES: Conceptualization, Data curation, Formal analysis, Funding acquisition, Investigation, Visualization, Writing – original draft, Writing – review & editing. AS: Data curation, Formal analysis, Investigation, Writing – review & editing. AA: Investigation, Writing – review & editing. JO’B: Conceptualization, Funding acquisition, Project administration, Supervision, Writing – review & editing.

## Footnotes

1 Data can be viewed at https://www.opt.uh.edu/research/zebrafish/.
