# Supplementary material for: Cost-effective strategies to knock down genes of interest in the retinas of adult zebrafish
Source: Front Cell Neurosci. 2024 Jan 23;17:1321337. doi: 10.3389/fncel.2023.1321337 (PMC10845135; doi:10.3389/fncel.2023.1321337)
Supplement: Supplementary file 2 [file Data_Sheet_1.docx]

Supplementary Material

Cost-effective strategies to knock down genes of interest in the retinas of adult Zebrafish

Eyad Shihabeddin, Abirami Santhanam, Alexandra L. Aronowitz, John O’Brien

*** Correspondence:**John O’Brien
[jobrien3@central.uh.edu](mailto:jobrien3@central.uh.edu)

Keywords: gene knockdowns, Vivo-Morpholino, siRNA, zebrafish retina, retinal degeneration, regeneration, photoreceptor, Retinitis Pigmentosa

# Supplementary Tables

**Table S1. qPCR run information.**

| Fluor | Target | Replicates | Sample | Biological Set Name | Cq | Cq Mean | Cq Std. Dev |
| --- | --- | --- | --- | --- | --- | --- | --- |
| SYBR | PCNA4 | Unkn-01 | 200 | PCNA siRNA | 25.04 | 24.94 | 0.080 |
| SYBR | PCNA4 | Unkn-01 | 200 | PCNA siRNA | 24.89 | 24.94 | 0.080 |
| SYBR | PCNA4 | Unkn-01 | 200 | PCNA siRNA | 24.90 | 24.94 | 0.080 |
| SYBR | PCNA4 | Unkn-02 | 201 | PCNA siRNA | 25.25 | 25.28 | 0.058 |
| SYBR | PCNA4 | Unkn-02 | 201 | PCNA siRNA | 25.34 | 25.28 | 0.058 |
| SYBR | PCNA4 | Unkn-02 | 201 | PCNA siRNA | 25.23 | 25.28 | 0.058 |
| SYBR | PCNA4 | Unkn-04 | 203 | PCNA siRNA | 26.68 | 26.63 | 0.049 |
| SYBR | PCNA4 | Unkn-04 | 203 | PCNA siRNA | 26.58 | 26.63 | 0.049 |
| SYBR | PCNA4 | Unkn-04 | 203 | PCNA siRNA | 26.61 | 26.63 | 0.049 |
| SYBR | PCNA4 | Unkn-05 | 204 | EGFP siRNA | 27.63 | 27.64 | 0.115 |
| SYBR | PCNA4 | Unkn-05 | 204 | EGFP siRNA | 27.54 | 27.64 | 0.115 |
| SYBR | PCNA4 | Unkn-05 | 204 | EGFP siRNA | 27.77 | 27.64 | 0.115 |
| SYBR | PCNA4 | Unkn-06 | 205 | EGFP siRNA | 26.47 | 26.48 | 0.022 |
| SYBR | PCNA4 | Unkn-06 | 205 | EGFP siRNA | 26.47 | 26.48 | 0.022 |
| SYBR | PCNA4 | Unkn-06 | 205 | EGFP siRNA | 26.51 | 26.48 | 0.022 |
| SYBR | PCNA4 | Unkn-07 | 206 | EGFP siRNA | 24.94 | 25.00 | 0.173 |
| SYBR | PCNA4 | Unkn-07 | 206 | EGFP siRNA | 24.86 | 25.00 | 0.173 |
| SYBR | PCNA4 | Unkn-07 | 206 | EGFP siRNA | 25.19 | 25.00 | 0.173 |
| SYBR | PCNA4 | Unkn-08 | 207 | EGFP siRNA | 23.39 | 23.29 | 0.087 |
| SYBR | PCNA4 | Unkn-08 | 207 | EGFP siRNA | 23.23 | 23.29 | 0.087 |
| SYBR | PCNA4 | Unkn-08 | 207 | EGFP siRNA | 23.24 | 23.29 | 0.087 |
| SYBR | PCNA4 | NTC | NTC |  | 36.94 | 36.94 | 0.000 |
| SYBR | PCNA4 | NTC | NTC |  |  | 0.00 | 0.000 |
| SYBR | PCNA4 | NTC | NTC |  | 39.32 | 39.32 | 0.000 |
| SYBR | GAPDH | Unkn-10 | 200 | PCNA siRNA | 22.08 | 21.96 | 0.106 |
| SYBR | GAPDH | Unkn-10 | 200 | PCNA siRNA | 21.87 | 21.96 | 0.106 |
| SYBR | GAPDH | Unkn-10 | 200 | PCNA siRNA | 21.94 | 21.96 | 0.106 |
| SYBR | GAPDH | Unkn-11 | 201 | PCNA siRNA | 21.34 | 22.06 | 0.481 |
| SYBR | GAPDH | Unkn-11 | 201 | PCNA siRNA | 22.32 | 22.06 | 0.481 |
| SYBR | GAPDH | Unkn-11 | 201 | PCNA siRNA | 22.28 | 22.06 | 0.481 |
| SYBR | GAPDH | Unkn-11 | 201 | PCNA siRNA | 22.29 | 22.06 | 0.481 |
| SYBR | GAPDH | Unkn-13 | 203 | PCNA siRNA | 23.97 | 23.83 | 0.160 |
| SYBR | GAPDH | Unkn-13 | 203 | PCNA siRNA | 23.88 | 23.83 | 0.160 |
| SYBR | GAPDH | Unkn-13 | 203 | PCNA siRNA | 23.66 | 23.83 | 0.160 |
| SYBR | GAPDH | Unkn-14 | 204 | EGFP siRNA | 25.54 | 25.56 | 0.059 |
| SYBR | GAPDH | Unkn-14 | 204 | EGFP siRNA | 25.62 | 25.56 | 0.059 |
| SYBR | GAPDH | Unkn-14 | 204 | EGFP siRNA | 25.51 | 25.56 | 0.059 |
| SYBR | GAPDH | Unkn-15 | 205 | EGFP siRNA | 25.52 | 25.06 | 0.398 |
| SYBR | GAPDH | Unkn-15 | 205 | EGFP siRNA | 24.86 | 25.06 | 0.398 |
| SYBR | GAPDH | Unkn-15 | 205 | EGFP siRNA | 24.80 | 25.06 | 0.398 |
| SYBR | GAPDH | Unkn-16 | 206 | EGFP siRNA | 23.13 | 23.19 | 0.050 |
| SYBR | GAPDH | Unkn-16 | 206 | EGFP siRNA | 23.22 | 23.19 | 0.050 |
| SYBR | GAPDH | Unkn-16 | 206 | EGFP siRNA | 23.20 | 23.19 | 0.050 |
| SYBR | GAPDH | Unkn-17 | 207 | EGFP siRNA | 20.66 | 20.48 | 0.156 |
| SYBR | GAPDH | Unkn-17 | 207 | EGFP siRNA | 20.39 | 20.48 | 0.156 |
| SYBR | GAPDH | Unkn-17 | 207 | EGFP siRNA | 20.40 | 20.48 | 0.156 |
| SYBR | GAPDH | NTC | NTC |  | 34.31 | 34.31 | 0.000 |
| SYBR | GAPDH | NTC | NTC |  | 36.82 | 36.82 | 0.000 |
| SYBR | GAPDH | NTC | NTC |  |  | 0.00 | 0.000 |

# Supplementary Movie

**Movie 1.** Video of intravitreal injection into an anesthetized adult zebrafish. Video depicts removal of vitreous with a blunt-tipped syringe followed by injection of Vivo-Morpholino oligo.
